# UV-C Irradiation Effectiveness on Mpox-Virus-Contaminated Surfaces

**DOI:** 10.3390/pathogens15010078

**Published:** 2026-01-10

**Authors:** Anna Gidari, Samuele Sabbatini, Carlo Pallotto, Sabrina Bastianelli, Sara Pierucci, Chiara Busti, Giulia Proietti, Alessia Lai, Giuseppe Vittorio De Socio, Daniela Francisci

**Affiliations:** 1 Department of Medicine and Surgery, Clinic of Infectious Diseases, “Santa Maria della Misericordia” Hospital, University of Perugia, 06132 Perugia, Italy; annagidari91@gmail.com (A.G.); sabrina.bastianelli@unipg.it (S.B.); sara.pierucci@unipg.it (S.P.); chiarabusti93@gmail.com (C.B.); giuliaproietti17@yahoo.it (G.P.); daniela.francisci@unipg.it (D.F.); 2 Department of Life Sciences, Health and Health Professions, Link Campus University, 00165 Rome, Italy; s.sabbatini@unilink.it; 3 Department of Biomedical and Clinical Sciences, University of Milan, 20122 Milan, Italy; alessia.lai@unimi.it; 4 Laboratory of Medical Microbiology and Virology, University of Insubria, 21100 Varese, Italy

**Keywords:** Mpox, UV-C, surfaces, glass, steel, plastic

## Abstract

Introduction: Mpox virus (MpoxV), an emerging zoonotic pathogen, has recently caused global concern due to increasing outbreaks beyond its traditional endemic regions. While transmission primarily occurs via close contact, fomites are also suspected of contributing. This study aims to evaluate the effectiveness of UV-C irradiation on MpoxV-contaminated surfaces. Methods: the virucidal activity of UV-C (254 nm) irradiation on MpoxV applied to plastic, glass, and stainless-steel surfaces was assessed. Using a viral stock of 2.49 × 10^5^ TCID_50_/mL, the samples were exposed to increasing UV-C doses. Viral titers were quantified through TCID_50_ and plaque assays. Results: A UV-C dose of 6.34 mJ/cm^2^ achieved a >2-log reduction of viral load, below the detection limit (31.6 TCID50/mL), on all tested surfaces. EC_90_ values were determined as 3.33 mJ/cm^2^ (plastic), 0.81 mJ/cm^2^ (stainless steel), and 1.98 mJ/cm^2^ (glass). No viable virus was detectable post-treatment at these doses on plastic and stainless steel while the titer was significantly reduced on glass. Conclusions: UV-C irradiation at low doses effectively inactivated MpoxV on various fomites. These findings support UV-C as a rapid and effective environmental disinfection strategy in healthcare and community settings to prevent indirect transmission of MpoxV.

## 1. Introduction

Mpox is a zoonotic infection caused by Mpox virus (MpoxV), family *Poxviridae*, subfamily *Chordopoxvirinae*, genus *Orthopoxvirus*, species *Orthopoxvirus monkeypox*, with two distinct clades (Clade 1 and clade 2) [1]. MpoxV, since the detection of the first human case in a child in the Democratic Republic of the Congo in 1970, historically caused sporadic outbreaks primarily confined to Central and West Africa [2]. On 23 July 2022, due to the global spread of Clade 2 MpoxV outside its usual geographic distribution in African countries, involving more than 100 countries across Europe and America, World Health Organization (WHO) declared it a Public Health Emergency of International Concern [3,4]. In particular, since the identification of MpoxV transmission outside endemic regions in May 2022, a large multi-country outbreak has been ongoing globally, with 153,961 cases and 380 deaths reported across 137 countries in all six (WHO) regions as of 30 June 2025 [5].

After the 2022 outbreak, the incidence of the infection outside Africa was significantly reduced. However, despite the decline in cases in Africa during 2023 and 2024, the first half of 2025 saw an approximately 50% increase in reported cases compared to the previous year [5].

Clinically, Mpox manifests with fever, lymphadenopathy, and distinctive vesiculopustular lesions resembling smallpox, albeit generally with lower mortality [6].

Transmission primarily occurs through direct contact with infected individuals or bodily fluids. In particular, the 2022 global outbreak has been associated with close intimate contact like during sexual activity, and most cases have been diagnosed among men who have sex with men. It is also reported a human-to-human transmission of mpox with respiratory droplets through a prolonged face-to-face contact [6].

However, indirect transmission via contaminated surfaces (fomites) plays a potentially critical role in virus spread, influenced by environmental factors such as temperature, humidity, and pH [6]. Prior studies demonstrated prolonged viral stability under low humidity and moderate temperatures, emphasizing the importance of environmental hygiene measures [7,8].

Ultraviolet-C (UV-C) irradiation at approximately 254 nm wavelength is widely recognized for its virucidal properties, effectively disrupting viral nucleic acids and halting replication processes [9]. UV-C has been successfully employed against various pathogens, including SARS-CoV-2, highlighting its potential utility in environmental disinfection [9,10].

This study aims to evaluate UV-C efficacy against MpoxV on different surfaces, determining the minimum UV-C dose needed to reduce viral titer below detectable limits.

## 2. Materials and Methods

### 2.1. Mpox Strain Isolation and Stock Preparation

All experiments were conducted using an *Orthopoxvirus monkeypox* (MpoxV) strain isolated in the Biosafety Level 3 (BSL-3) Virology Laboratory at the Clinic of Infectious Diseases, University of Perugia—Santa Maria della Misericordia Hospital, Perugia, Italy.

For all the experiment, a strain isolated from a symptomatic patient admitted to the Clinic of Infectious Diseases of the same hospital was used, as described previously [11]. Briefly, following the incision of a vesicle, a swab was collected, and the diagnosis was confirmed by a PCR test. Subsequently, transport medium (UTM) was incubated with a 1:1 Eagle’s minimum essential medium (MEM) supplemented with penicillin–streptomycin (1%) and left to react for 1 h at 4 °C to reduce bacterial contamination. The resulting suspension was inoculated onto a monolayer of Vero E6 cells and incubated for 2 h at 37 °C with 5% CO_2_ atmosphere. Following this initial incubation, the medium was replaced with MEM supplemented with 1% fetal bovine serum (FBS) at 37 °C with 5% CO_2_. The plates were checked every 24 h to detect the cytopathic effect (CPE). After CPE appearance, supernatant was recovered, filtered (filter 0.45 µm) and viral titer was determined by Median Tissue Culture Infectious Dose (TCID_50_) endpoint dilution assay [12] and stock aliquots were stored at −80 °C. The viral stock had a titer of 2.49 × 10^5^ TCID_50_/mL and aliquots stored frozen were thawed immediately before use in each experiment.

### 2.2. MpoxV Strain Sequencing

The MpoxV strain was sequenced at the Laboratorio di Malattie Infettive, Department of Biomedical and Clinical Sciences, University of Milan. The strain was identified as Clade IIb lineage C.1.1.

Briefly, DNA was extracted from sample using the QIAamp DNA Blood kit (QIAGEN, Hilden, Germany), fragmented using a Covaris M220 ultrasonicator (Covaris, Woburn, MA, USA) and checked using a 4200 TapeStation System (Agilent Technologies, Santa Clara, CA, USA) to verify dimensions. The libraries were prepared using the Nextera XT DNA Library Preparation Kit (Illumina, San Diego, CA, USA) and sequencing was performed using a Miseq sequencer (Illumina, San Diego, CA, USA) with 300 cycles.

Reads were mapped to a reference genome sequences (MT903343.1) using Geneious software V.11 (Biomatters, Auckland, New Zealand) (http://www.geneious.com) obtaining an average depth of 18.5× (min 1–max 1731). Clade assignment was performed using NextClade (https://clades.nextstrain.org/).

### 2.3. Inanimate Surfaces

The materials selected for testing were plastic (polystyrene, 24-well plates; Corning, Falcon^®^, New York, NY, USA), glass (sterile disks, 12 mm diameter, Corning, Falcon^®^, New York, NY, USA), and stainless steel (AISI 304 sterile disks, 12 mm diameter, Promagroup, Umbertide, Perugia, Italy). The glass and stainless-steel disks were sterilized by autoclaving and subsequently placed into 24-well plates [10].

### 2.4. UV-C Irradiation Assay

All the experiments were conducted in a BSL-3 laboratory as previously described [10]. Each experiment was performed in triplicate and independently repeated at least two or three times for each type of material. Ambient temperature and relative humidity were continuously monitored and maintained at approximately 23–25 °C and 40–50%, respectively.

To assess the virus recovery efficiency from tested surfaces, two separate aliquots of 10 µL virus suspension were used: one aliquot was immediately processed to determine the viral titer by TCID_50_ assay, while the second aliquot was deposited onto the surface and recovered by washing after a 30 min incubation period (T_0_) through washing. Recovery efficiency was then calculated using the following formula: [TCID_50_/mL recovered virus (T_0_)/TCID_50_/mL virus aliquot] × 100 [10].

Given the viral stock (2.49 × 10^5^ TCID_50_/mL), 10 or 50 μL of frozen viral stock were placed on different surfaces of the materials by a sterile pipet tip.

A monochromatic UV-C lamp emitting at 254 nm, with an irradiance of 0.82 mW/cm^2^, was placed at 30 cm from the different surfaces. The UV light dose was measured by the manufacturer (Bazzica Engineering^®^, Trevi, Italy), who also produced and provided the lamp. Measurements were performed using a photometer (RMD Sensor UVC 200–280 nm, 0–10 W/cm^2^; Opsytec Dr. Gröbel GmbH, Ettlingen, Germany) at the same 30 cm distance used in the experiments.

Starting from a dose of 136.81 mJ/cm^2^, corresponding to 180 s of exposure, decreasing doses of UV-C were applied by reducing the exposure time, in order to identify the minimum dose capable of reducing the viral titer below the detection limit of the method (31.6 TCID_50_/mL) corresponding to a >2 Log reduction. Simultaneously, a control plate was maintained under identical conditions but shielded with aluminium foil (shielded plate). Additional plates containing only culture medium were also exposed under the same environmental conditions [10].

Following UV-C exposure, supernatants were recovered and titers were determined as TCID_50_/mL [10]. Once the lowest UV-C dose capable of reducing the viral load below the detection limit was identified, the supernatant from the corresponding sample was further analysed using a plaque assay. Plaque assay has been performed as previously described [13,14].

### 2.5. Statistical Analysis

Statistical analysis was performed using Graphpad Prism 8.31 (San Diego, CA, USA). Kolmogorov–Smirnov test was used to test data normality. Based on this test, mean with the respective standard deviation (SD) or median with interquartile range (IQR) were used to present data. EC_50_, and EC_90_ concentrations were calculated using three-parameter regression modelling.

## 3. Results

The mean recovery efficiency was 49%.

Several experiments were conducted on plastic surfaces using different time points, and thus varying UV-C doses, to generate a dose–response curve. Subsequently, four distinct UV-C doses were tested on each material.

**Figure 1:**
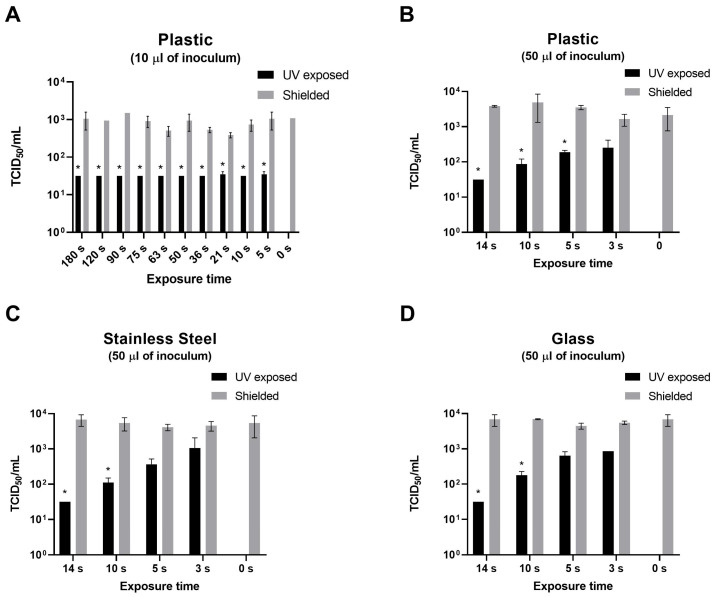
Effect of UV-C (254 nm) exposure on simulated MpoxV surface contamination. Ten or fifty microliters of viral stock were applied to various materials ((**A**,**B**), Plastic; (**C**), Stainless Steel; (**D**), Glass); and either recovered after 30 min (T_0_) or exposed to different UV-C doses (black bars). A polystyrene plate shielded with aluminium foil served as the control for each exposure time (grey bars, “Shielded”). Viral titers (TCID_50_/mL) were determined from supernatants recovered from each surface. No significant differences were observed between shielded controls and T0. * *p* < 0.05, UV-C treated materials vs. UV-C shielded.

As shown in Figure 1A,B, no significant differences were observed between T_0_ and shielded plates, nor among shielded plates across the different exposure times (3–180 s). Similar results were obtained for stainless steel and glass over the range of 0–14 s (Figure 1C,D).

UV-C doses tested on plastic were: 1.24 mJ/cm^2^ (3 s), 2.07 mJ/cm^2^ (5 s), 4.33 mJ/cm^2^ (10 s), 6.34 mJ/cm^2^ (14 s), 10.25 mJ/cm^2^ (21 s), 20.06 mJ/cm^2^ (36 s), 30.6 mJ/cm^2^ (50 s), 40.95 mJ/cm^2^ (63 s), 50.71 mJ/cm^2^ (75 s), 63.01 mJ/cm^2^ (90 s), 87.61 mJ/cm^2^ (120 s), 136.81 mJ/cm^2^ (180 s).

As shown in Figure 1A, UV-C exposure was first tested on a low viral load, corresponding to 10 µL of viral stock (2.49 × 10^3^ TCID_50_). All UV-C doses, with the exception of the lowest one (2.07 mJ/cm^2^, 5 s), were effective in reducing the viral titer below the detection limit of the method. The 2.07 mJ/cm^2^ dose reduced the titer to 83.1 TCID_50_/mL (SD, 67.8 TCID_50_/mL). In this instance, it was not possible to generate a complete dose–response curve, and therefore the lowest dose (1.24 mJ/cm^2^, 3 s) was not tested.

**Figure 2:**
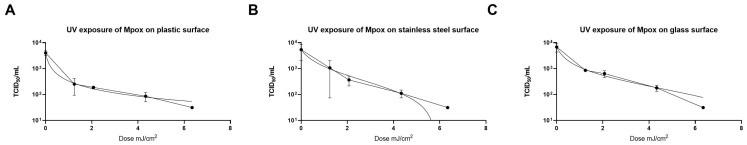
Effect of UV-C (254 nm) exposure on simulated Mpox surface contamination. Fifty microliters of viral stock were applied to different materials and either recovered after 30 min (T0) or exposed to graded UV-C doses. A polystyrene plate covered with aluminium foil was used as a control. Viral titers (TCID_50_/mL) were determined from recovered supernatants. Dose–response curves are shown for plastic (**A**), stainless steel (**B**), and glass (**C**). Data represent the mean ± standard deviation of two or three independent experiments. UV-C dose is expressed in mJ/cm^2^.

As shown in Figure 2, UV-C exposure was tested on 1.25 × 10^4^ TCID_50_ of virus, corresponding to 50 µL of viral stock. A dose of 6.34 mJ/cm^2^ was the lowest dose that reduced the viral titer below the detection limit of the method (31.6 TCID_50_/mL) corresponding > 2 Log reduction. Lower doses were subsequently tested to generate a dose–response curve, and the data were analysed using a three-parameter linear regression model. The UV-C treatment showed a half-maximal effective concentration (EC_50_) of 0.09 mJ/cm^2^ (95% confidence interval, CI, lower value not available, NA, to 0.33) and an EC_90_ of 3.33 mJ/cm^2^ (Figure 2A). Although an EC_50_ best-fit value was obtained, it was not possible to calculate a complete confidence interval due to the very low EC_50_ value.

Comparable results were observed for stainless steel and glass. For both materials, the dose of 6.34 mJ/cm^2^ was the minimum required to reduce the viral titer below the detection threshold (31.6 TCID_50_/mL). As shown in Figure 2B, the UV-C treatment of Mpox on stainless steel yielded an EC_50_ of 0.37 mJ/cm2 (95% CI NA, to 4.2) and an EC_90_ of 0.81 mJ/cm^2^. For glass, the EC_50_ of UV-C on MpoxV was 0.22 (95% CI NA–1.17) and the EC_90_ was 1.98 mJ/cm^2^ (Figure 2C).

**Figure 3:**
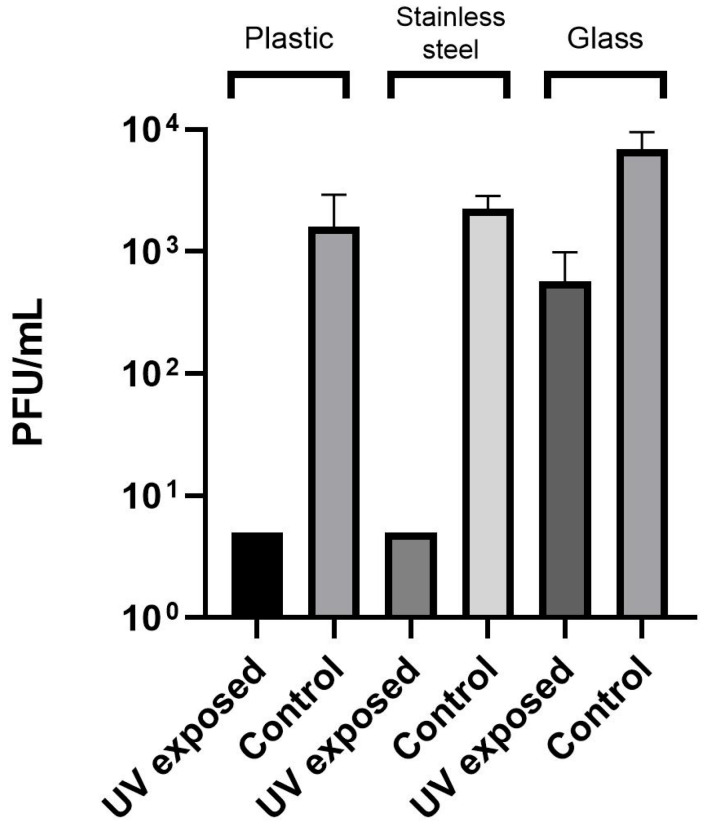
Eradication of Mpox virus from contaminated surfaces. A UV-C dose of 6.34 mJ/cm^2^ was the lowest dose that reduced viral titers below the detection limit of the assay (31.6 TCID_50_/mL). To assess whether this dose achieved complete viral inactivation, samples treated with 6.34 mJ/cm^2^ and corresponding controls were recovered and titrated using plaque assay (PFU/mL). This UV-C dose completely inactivated MpoxV on plastic and stainless steel and significantly reduced viral titers on glass. Data represent the mean ± standard deviation of two or three independent experiments.

Furthermore, to verify viral eradication, samples exposed to a UV-C dose of 6.34 mJ/cm^2^, along with their corresponding control wells, were recovered and titrated using a plaque assay, with results expressed as PFU/mL. As shown in Figure 3, the UV-C dose 6.34 mJ/cm^2^ eradicated MpoxV on plastic and stainless steel, while it resulted in a significant reduction of viral titer on glass.

## 4. Discussion

MpoxV transmission via contaminated surfaces remains a significant public health concern, particularly given its environmental stability under favorable conditions (low humidity, moderate temperature). MpoxV has been shown to remain stable for more than 49 days at 4 °C, and up to 42 days at 37 °C or at room temperature [15]. A recent study explored viral persistence on various fomites, demonstrating that infectious MpoxV can persist for up to 21 days on non-porous surfaces at low temperatures. In contrast, porous materials such as cotton exhibited a rapid loss of infectivity, particularly at room temperature [8]. The MpoxV persistence in wastewater could also be an important issue, especially in low-income countries [7].

These findings underscore the need for effective disinfection strategies in outbreak control.

UV-C irradiation is a well-established method for microbial inactivation and has demonstrated effectiveness against a wide range of bacteria and viruses, including SARS-CoV-2. Raeiszadeh et al. reviewed the application of UV-C for disinfection and highlighted its effectiveness against SARS-CoV-2 and other coronaviruses, with doses ranging from 1.2 to 40 mJ/cm^2^ [16]. Buonanno et al. effectively inactivated airborne human coronaviruses (HCoV-229E, HCoV-OC43) using far-UV-C at doses around 1–2 mJ/cm^2^, suitable for continuous disinfection in occupied spaces [17]. Heilingloh et al. reported a 1-log reduction in SARS-CoV-2 titers at an exposure dose of approximately 292 mJ/cm^2^ [18]. Our group previously demonstrated effective SARS-CoV-2 inactivation at relatively low UV-C doses (10.25–23.71 mJ/cm^2^) on plastic, glass, and stainless-steel surfaces [10]. While coronaviruses are among the most extensively studied pathogens in the context of UV-C disinfection due to their global health impact, the efficacy of UV-C has also been tested on a variety of other viruses. Gerba et al. achieved three-log titer reductions of echovirus 1, echovirus 11, coxsackievirus B3, coxsackievirus B5 and poliovirus 1 using doses of 25, 20.5, 24.5, 27, and 23 mW/cm^2^, respectively. In the same study, human adenovirus type 2 was found to be more resistant, requiring a dose of 119 mW/cm^2^ for 99.9% inactivation [19]. In addition, a recent research has demonstrated the effectiveness of UV-C irradiation in decontaminating environmental surfaces contaminated with Marburg virus [20].

Collectively, these data underscore the broad-spectrum effectiveness of UV-C light for pathogen inactivation on fomites.

In a recent study, Mariotti et al. specifically investigated UV-C inactivation of MpoxV, demonstrating complete virus inactivation after 15 min of exposure; however, the precise UV-C dose applied was not reported [21]. Our findings significantly expand these observations, by establishing a much lower effective UV-C dose (6.34 mJ/cm^2^) for MpoxV inactivation, with EC_90_ values varying according to surface type. This dose is notably lower than previously suggested, reinforcing the practicality of UV-C as a disinfection method suitable for routine use.

The practical implications of these findings are directly relevant to public and healthcare environments. Mobile or fixed UV-C systems could be employed to rapidly disinfect high-touch surfaces in locations such as public transportation, educational facilities, healthcare settings, and retail environments. Implementation of such systems could play a critical role in limiting MpoxV transmission in community settings.

Nevertheless, potential concerns related to accidental overexposure to UV-C lights must be addressed.

A recent study evaluated the benefits of UV-C-based air disinfection in aircraft in relation to the potential risks of UV-C overexposure for passengers and crew. The authors demonstrate that the risks are significantly lower than the benefits and no-long term effects are expected [22].

Limitations of this study include potential discrepancies between laboratory-controlled experiments and real-world settings, and variability in contamination levels and organic load on surfaces. Further research is required to evaluate UV-C effectiveness under practical conditions.

## 5. Conclusions

UV-C irradiation effectively inactivates MpoxV on various surfaces at low doses. These results advocate broader implementation of UV-C protocols in healthcare and public settings to mitigate MpoxV environmental transmission risks.

## Data Availability

The data presented in this study are available on request from the corresponding author.
